# Effect of Cryogenic Treatment on Internal Residual Stresses of Hydrogen-Resistant Steel

**DOI:** 10.3390/mi12101179

**Published:** 2021-09-29

**Authors:** Fengxiang Shang, Jinxing Kong, Dongxing Du, Zheng Zhang, Yunhua Li

**Affiliations:** Institute of Mechanical Manufacturing Technology, China Academy of Engineering Physics, Mianyang 621900, China; bernard.sfx@gmail.com (F.S.); caep-zhengzhang@hotmail.com (Z.Z.); liyunhua19@gscaep.ac.cn (Y.L.)

**Keywords:** hydrogen-resistant steel, cryogenic treatment, contour method, residual stress, grain refinement

## Abstract

To reduce the influence of internal residual stress on the processing deformation of thin-walled hydrogen-resistant steel components, combined aging cryogenic and high-temperature treatment was used to eliminate the residual stress, and the effect of cryogenic process parameters on the initial residual stress of the specimens was compared and analyzed based on the contour method. X-ray diffraction, electron backscatter diffraction, and transmission electron microscopy were used to research the mechanism of the effect of cryogenic treatment on the internal residual stress of the specimen. After forging, the internal residual stress distribution of the hydrogen-resistant steel specimens without aging was characterized by tensile stress on the core and compressive stress on both sides, with a stress amplitude of −350–270 MPa. After compound treatment of −130 °C for 10 h and 350 °C for 2 h, the internal residual stress distribution remained unchanged, and the stresses decreased to −150–100 MPa. The internal residual stresses were reduced by 57–63% compared with the untreated specimens. The cryogenic treatment did not cause phase transformation and carbide precipitation of the hydrogen-resistant steel material. Instead, grain refinement and dislocation density depletion were the main reasons for the reduction in internal residual stresses in the specimens.

## 1. Introduction

Thin-walled components are characterized by their light weight, high specific strength, and integrated nature, which are used extensively in aviation, aerospace, weapons, and other industrial fields [1]. Due to a large amount of material removal and the strong effect of internal residual stress redistribution during manufacturing, this component type easily undergoes machining deformation. The internal residual stress of the material is an important factor that causes machining deformation of thin-walled components [2,3]. To reduce the influence of internal residual stress on the machining deformation of thin-walled components, mechanical tension [4], natural aging [5], heat treatment aging [6], vibration aging [7], and cryogenic treatment [8,9,10] methods are used extensively in research to reduce or eliminate internal residual stress. Cryogenic treatment has been used extensively in production because of its obvious stress relief effect and unlimited size and shape of parts [11].

For example, Wei et al. [8] used the X-ray diffraction method to explore the effect of cryogenic treatment on the residual stress of 35 MnB alloy steel. The residual stress of the specimen was reduced from 46 to −220 MPa, which shows that the residual stress on the 35 MnB alloy steel surface can be reduced. Retained austenite distributed at the martensite crystal boundary can relax the stress concentration caused by the accumulation of dislocations, and thereby the residual stress inside the structure is reduced. Senthilkumar et al. [9] used X-ray diffraction to explore the effects of cryogenic treatment and a tempering sequence at −80 °C and −196 °C on the residual stress of 4140 steel. A lower cryogenic temperature was more conducive to the formation of residual compressive stress. Tempering after cryogenic treatment reduced the internal residual stress of the specimens. Cryogenic treatment reduced the thermodynamic stability of the martensite and promoted the diffusion and segregation of carbon atoms, and thus carbide precipitation reduced the residual stress of the specimens. Tempering after cryogenic treatment destroys the martensite structure and reduces the residual stress. Weng et al. [10] used the drilling method to explore the effect of cryogenic treatment on the internal residual stress of 7050 aluminum alloy, and the result showed that cryogenic treatment can homogenize and release the residual stress of the 7050 aluminum alloy effectively. Cryogenic treatment can contract the crystal lattice, which can induce micro-plastic deformation. Therefore, the uneven residual stress can be eliminated and homogenized after cryogenic treatment via micro-plastic deformation.

In summary, cryogenic treatment has been used to reduce the internal residual stresses of specimens with drilling and X-ray diffraction to evaluate the internal residual stresses before and after cryogenic treatment. As the mechanism of cryogenic treatment to reduce residual stresses in different materials requires study, drilling can only measure the residual stresses at limited points on the specimens, and X-ray diffraction can only measure residual stresses at the specimen surface. To obtain more information about the internal residual stresses in materials, in 2001, Prime et al. [12] proposed the contour method based on the principle of elastic superposition to evaluate the internal residual stresses of specimens. This process is simple, has economic applicability and insensitivity to the microstructure [13], and can provide the complete cross-sectional stress distribution at multiple sample positions. 

Therefore, this work focused on studying the thin-walled components of hydrogen-resistant steel that are used in nuclear energy and weapons. After solution treatment, large internal residual stresses and serious material processing deformation resulted. When the material is heated between 480 and 950 °C, chromium-containing carbides precipitate, which affects the mechanical properties and intergranular corrosion resistance [14]. Therefore, material aging is usually carried out at 350 °C, but it is difficult to reduce the internal residual stress. To achieve high-precision processing of hydrogen-resistant steel thin-walled components, this work introduced cryogenic aging treatment based on conventional aging heat treatment to reduce the internal residual stress. The effect of cryogenic aging was measured and evaluated based on the contour method, combined with X-ray diffraction (XRD), electron backscatter diffraction (EBSD), and transmission electron microscopy (TEM), to explore the mechanism of the effect of cryogenic treatment on the internal residual stresses of hydrogen-resistant steel materials. This research provides technical support for deformation control of hydrogen-resistant steel thin-walled components.

## 2. Experimental Process

### 2.1. Test Material

Hydrogen-resistant steel was chosen as the test material, with the chemical composition as listed in Table 1. The original rolled bar (75 mm diameter) was heated at 900 °C and forged into a square of 105 mm × 105 mm × 18 mm. After 2 h of heat preservation at 1050 °C, solid solution treatment was carried out by water quenching, and the sample was milled into a specimen of 98 mm × 98 mm × 14 mm, ap = 0.2 mm, af = 0.03 mm/z, v = 280 m/min.

### 2.2. Experimental Scheme and Selection of Technological Parameters

**Figure 1:**
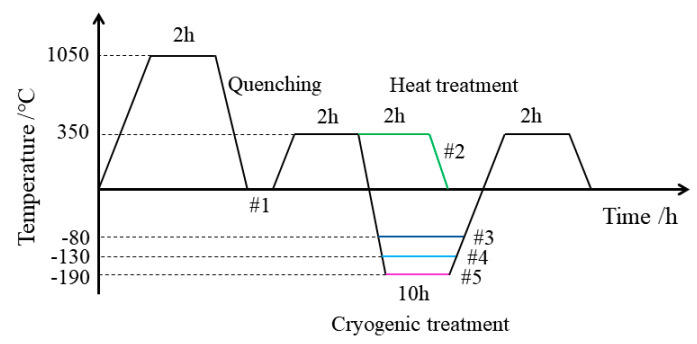
Schematic diagram of cryogenic treatment process.

Heat treatment of specimens was carried out in a muffle furnace (Nabertherm, Germany, model LT15/11/B410), the time for the sample to rise to 350 °C was set to 30 min, and cooling was conducted with the furnace. Cryogenic treatment was carried out in a cryogenic furnace (Shanli Company, model SL500) with liquid nitrogen as the medium, the cooling rate of the sample was 4 °C/min, and heating was conducted with the furnace. The processing parameters for different aging treatments are listed in Table 2 and Figure 1. Cryogenic compound high-temperature aging treatment for different parameters is referred to as cryogenic treatment in this paper.

According to the experimental design in Figure 1, specimen #1 was the untreated specimen after solid solution treatment. Specimen #2 was treated for 4 h of aging treatment at 350 °C, which is commonly applied in production. Cryogenic treatment was arranged between two conventional heat treatments to reduce the possibility of specimen cracking that is caused by direct cryogenic treatment, and the temperatures that were applied in cryogenic treatment were −80 °C, −130 °C, and −190 °C. The temperature and total holding time in the two heat treatments of specimens #3 to #5 were the same as for specimen #2. Three specimens were tested for each cryogenic treatment parameter.

### 2.3. Procedures to Measure Internal Residual Stress Based on the Contour Method

The internal residual stress of the specimens was measured by the contour method that was proposed by Prime et al. [12], and the following steps were used to measure and evaluate the internal residual stress. 

**Figure 2:**
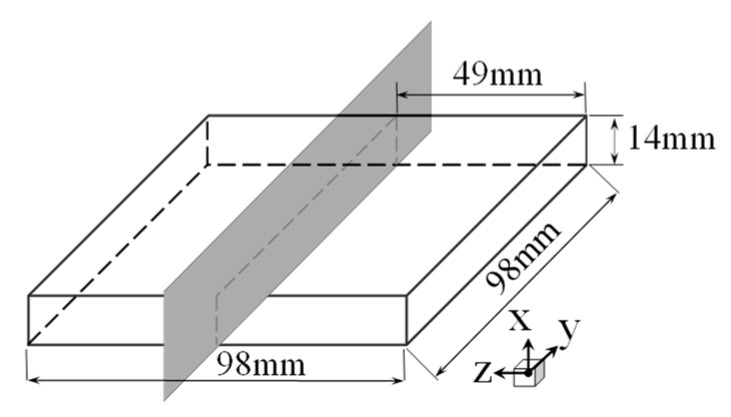
Schematic diagram of specimen size and cutting surface.

**Figure 3:**
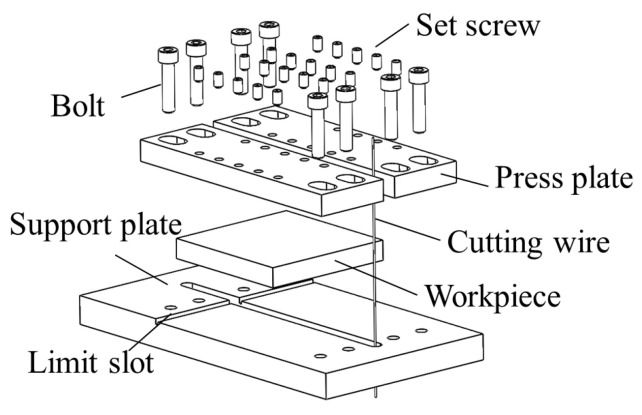
Schematic diagram of specimen cutting and clamping.

**Figure 4:**
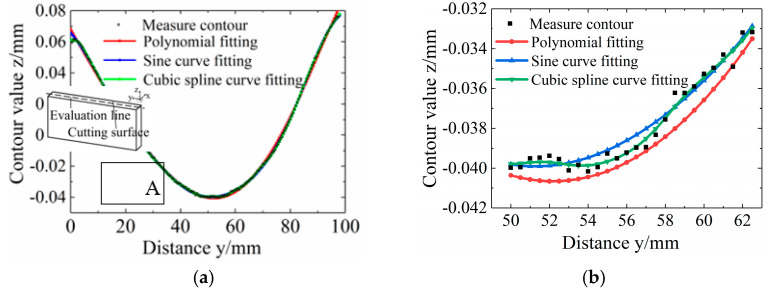
Fitted contour comparison of specimens. (**a**) Fitted contour comparison. (**b**) Amplifier of area A in Figure 4a.

(1) Cutting of the specimens. The specimens were cut into halves along the y-axis by a high-precision slow wire electrical discharge machining (EDM) wire cutting machine (Sodick AQ400LS), with an average cutting speed of 0.5 mm/min, as shown in Figure 2. Copper wire (0.025 mm diameter) was used for cutting. The specimen was clamped with a support and pressing plate, which were fixed with hexagonal cylindrical head screws and hexagonal set screws during cutting. A schematic of the specimen clamping is shown in Figure 3. 

(2) Measuring contour. High-precision contour-measuring equipment was used to measure the deformation contour of the cutting surface. A contact CMM (Zeiss New Contura Vastxt) with a test error of 1.5 + L/360 μm (L is the measurement length) and a step size of 0.2 mm was used to measure the contour of the cutting surface. 

(3) Data processing. The measured contour values of the two cutting surfaces were averaged and fitted to eliminate errors. Polynomial, sine, and cubic spline curves were applied for fitting. The fitting errors of the above three methods were compared and analyzed with the original data. As shown in Figure 4a, the midline of the specimen section was taken along the length direction as the evaluation line. The squared variances of polynomial fitting, sine curve fitting, and cubic spline curve fitting were 0.00158759, 0.00568631, and 0.000183585, respectively. Figure 4b shows the zoomed area in A of Figure 4a, from which it can be found that the curve from the cubic spline interpolation function had a better fit to the original measurement data. Therefore, the cubic spline interpolation function was chosen to fit the cutting surface contour.

(4) Stress reconstruction. The fitted contour value was applied as the displacement boundary condition based on the principle of elastic superposition, and a finite element model was developed by using the deformed contour in the reverse direction. A finite element model of the sample after cutting was established by ABAQUS software (ABAQUS Inc., Palo Alto, CA, USA), and the constraints imposed should not affect the free deformation of the contour. In finite element analysis, the material was set to be isotropic and homogeneous, with an elastic modulus of 206 GPa, and a Poisson ratio of ν = 0.28. The grid size was 0.5 mm × 0.5 mm × 0.5 mm, with 537,824 elements, and the grid type was a hexahedral eight-node reduced integral element (C3D8R). The distribution of residual stress perpendicular to the cutting surface was obtained by finite element analysis.

### 2.4. Test Instrument 

The phase was detected by XRD and an Xpert Pro X-ray diffractometer (Malvern Panalytical) to analyze the mechanism of cryogenic treatment on the internal residual stress. The specimen was 40 mm × 40 mm × 5 mm, and the 2θ ranged from 20° to 100° during the test. EBSD scanning electron microscopy (Zeiss ARUGA) was applied in the microstructural characterization of the specimens that were subjected to mechanical grinding, polishing, and vibration polishing. Channel 5 was used as the post-processing software. FEI Talos F200X TEM was used to observe the precipitate microstructure and morphology, and specimens prepared for TEM were obtained by conventional ion thinning.

## 3. Results and Discussion 

### 3.1. Results of Stress Reconstruction

**Figure 5:**
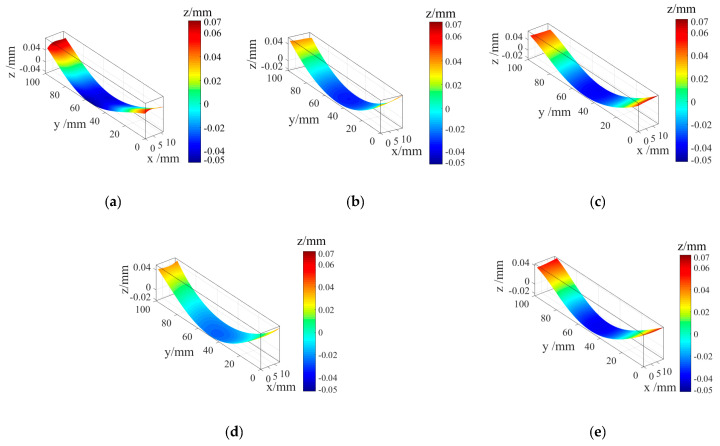
Fitting contour of specimens for different cryogenic parameters. (**a**) Specimen #1 fitting contour. (**b**) Specimen #2 fitting contour. (**c**) Specimen #3 fitting contour. (**d**) Specimen #2 fitting contour. (**e**) Specimen #2 fitting contour.

Figure 5 shows the contours of specimens #1 to #5 after cubic spline function fitting. The contour distribution of untreated specimen #1 was concave in the middle and convex on both sides, and the range of the contour amplitude was −0.05–0.07 mm. There was no obvious change in contour distribution characteristics of specimen #2 after the heat treatment and specimens #3 to #5 after the cryogenic treatment, whereas the contour amplitude decreased. The contour amplitude of specimens that underwent cryogenic treatment at 350 °C for 2 h, 130 °C for 10 h, and 350 °C for 2 h decreased to −0.02–0.04 mm.

**Figure 6:**
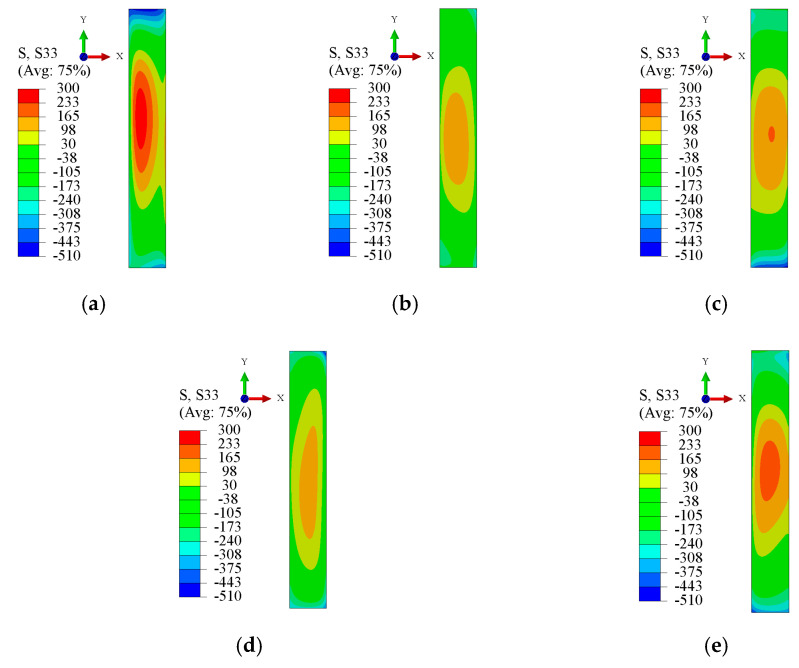
Stress reconstruction on cutting surface of specimens for different cryogenic parameters. (**a**) Specimen #1. (**b**) Specimen #2. (**c**) Specimen #3. (**d**) Specimen #4. (**e**) Specimen #5.

The finite element model was established according to the cut sample. The cutting surface of the sample was an XY cross-section. The stress that was obtained through stress reconstruction was perpendicular to the cross-section, that is, a Z-direction stress *σ*_z_. The specimen stress reconstruction for different aging treatment parameters is shown in Figure 6. The distribution of stress *σ*_z_ on the cutting surface of samples #1 to #5 was as follows: tensile stress on the core and compressive stress on both sides.

Compared with the stress distribution of untreated specimen #1, the stress gradients of specimen #2 after heat treatment and specimens #3 to #5 after cryogenic treatment were smaller. The peak tensile stress of untreated specimen #1 was 273 MPa, whereas the tensile stress peaks of specimens #2 to #5 after treatment were 148 MPa, 167 MPa, 127 MPa, and 194 MPa, respectively. The internal stress of the specimens after cryogenic treatment decreased significantly. The above results indicate that a lower cryogenic temperature did not result in a lower internal residual stress. There was an obvious inflection point where the cryogenic treatment had the best effect on reducing stress.

**Figure 7:**
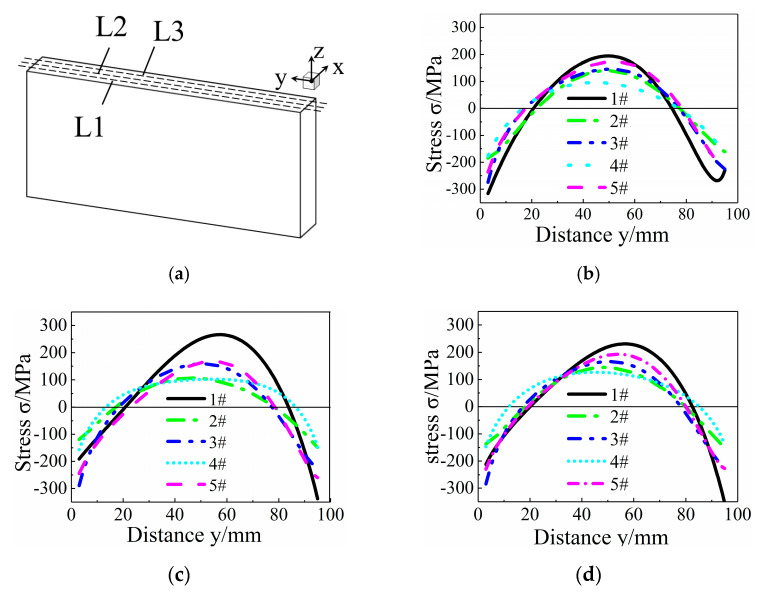
Comparison of stress along evaluation lines on cutting surface. (**a**) Schematic diagram of evaluation line. (**b**) Stress along line L1. (**c**) Stress along line L2. (**d**) Stress along line L3.

Three evaluation lines at the 25%, 50%, and 75% positions along the X-direction from the cutting surface were selected to compare the stress changes in specimens from each group. A schematic of the evaluation lines is shown in Figure 7a. The residual stress distribution on the three evaluation lines is shown in Figure 7. The stress distribution characteristics on L1, L2, and L3 remained unchanged, and they all showed a compressive stress on both sides and a tensile stress on the core. From Figure 7c, we can see that the maximum tensile stresses from #1 to #5 are 300 MPa, 100 MPa, 200 MPa, 100 MPa, and 200 MPa, respectively. There was a large stress in specimen #1, and the residual stresses of the specimens decreased specifically after heat and cryogenic treatment, in which the heat treatment at 350°C for 4 h and cryogenic treatment at 350 °C for 2 h, −130 °C for 10 h, and 350 °C for 2 h had a better effect on stress reduction. For the analysis of L2, the stress amplitude of specimen #1 was −350–270 MPa, and the stress amplitudes were −150–100 MPa after heat treatment at 350 °C for 4 h and cryogenic treatment at 350 °C for 2 h, −130 °C for 10 h, and 350 °C for 2 h, but the stress gradient was smaller in the core of the latter. For the L1 and L3 lines, the stress amplitudes were −300–200 MPa, −200–130 MPa, and −150–100 MPa, and the treatment conditions were untreated, heat treatment at 350 °C for 4 h, and cryogenic treatment at 350 °C for 2 h, −130 °C for 10 h, and 350 °C for 2 h, respectively. Single heat treatment and cryogenic treatment can reduce the internal residual stress, and the deduction in residual stress in the core of the specimen was more obvious according to line L2. In this test, the parameters of 350 °C for 2 h, −130 °C for 10 h, and 350 °C for 2 h showed the optimal stress relief effect among the four groups, and the stress amplitude decreased from −350–270 to −150–100 MPa.

### 3.2. Mechanism of the Effect of Cryogenic Treatment on Internal Residual Stress 

#### 3.2.1. Phase Transition Analysis

**Figure 8:**
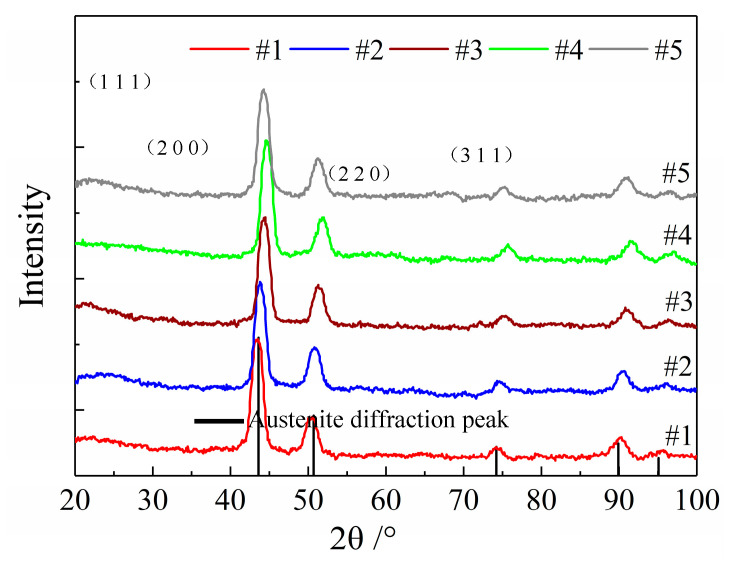
X-ray diffraction patterns of specimens for different cryogenic parameters.

The diffraction peaks from the treated specimen XRD patterns were well indexed without extra diffraction peaks for other phases and matched with the austenite diffraction peak pattern, which indicates that the hydrogen-resistant steel material was single-phase austenite, and the austenite crystal planes were (1 1 1), (2 0 0), (2 2 0), and (3 1 1), as shown in Figure 8. Therefore, it was concluded that the austenite phase of the hydrogen-resistant steel remained stable without phase transition after cryogenic treatment.

Zhao [15] believes that the occurrence of phase change requires a large degree of subcooling as a prerequisite. Therefore, phase transition must be accompanied by a continuous decrease in temperature, in order to obtain a large phase transition driving force. The lowest cryogenic temperature in this test was −190 °C, which was close to the limit temperature of the cryogenic medium (liquid nitrogen). If hydrogen-resistant steel specimens undergo phase transition after cryogenic treatment, then the driving force at this subcooling temperature is sufficient to create the phase transformation. Therefore, the absence of phase change in the hydrogen-resistant steel specimen is not due to insufficient undercooling.

**Figure 9:**
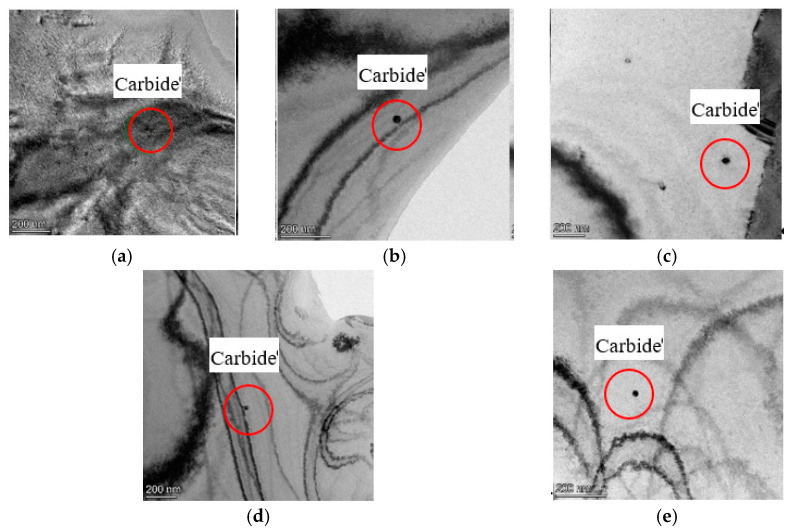
Morphology of precipitates for different cryogenic parameters. (**a**) Specimen #1. (**b**) Specimen #2. (**c**) Specimen #3. (**d**) Specimen #4. (**e**) Specimen #5.

In this work, carbide precipitation of the specimens was observed by TEM to verify whether the reduction in residual stress by cryogenics was related to carbide precipitation. The result supports the conclusion that no phase transformation of the hydrogen-resistant steel specimens occurred after cryogenic treatment because the carbide precipitation was closely related to the phase transformation. Figure 9 shows the TEM microstructural morphology of the specimens. Particles with obvious contrast are precipitated carbides. For 1000 × 1000 nm, there was no obvious carbide precipitation in specimens after cryogenic treatment. Therefore, the carbide content of the hydrogen-resistant steel specimens after cryogenic treatment was stable.

The transition of austenite to other phases caused volume changes, and carbon segregation because the internal carbon atoms diffused locally. When the furnace returned to room temperature, the carbon atoms broke through the grain boundaries and entered the carburized body, which resulted in carbide precipitation, which caused stress release within the material [16]. S. Zhirafar [17] believed that the cryogenic treatment caused the sample to undergo a phase change, accompanied by the precipitation of carbides during the tempering process. Das et al. [18] believed that cryogenic treatment increased dislocations and twin boundaries in the specimens, and that carbon atoms precipitated from the martensite matrix and formed fine carbides with alloying elements during tempering. Liu et al. [19] believed that martensitic stainless steels change the internal residual austenite content under different tempering conditions, which is closely related to the precipitation of carbides. As a result, carbide precipitation was closely related to the degree of phase transformation of the specimens. The X-ray diffraction patterns of the specimens indicate that the hydrogen-resistant steel did not undergo phase transformation after cryogenic treatment, meaning there was no significant precipitation of carbides because of phase transformation.

#### 3.2.2. Microstructural Analysis 

**Figure 10:**
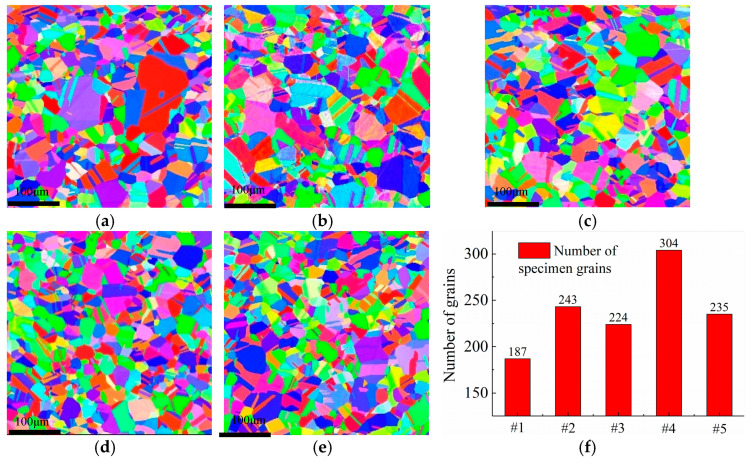
Grain morphology of specimens for different cryogenic parameters. (**a**) Specimen #1. (**b**) Specimen #2. (**c**) Specimen #3. (**d**) Specimen #4. (**e**) Specimen #5. (**f**) Number of grains for different cryogenic parameters.

Figure 10 shows the microstructural morphology of hydrogen-resistant steel specimens for different cryogenic treatment parameters measured by EBSD. The grains of specimen #1 were coarse and unevenly distributed, and there were 187 specimen crystal grains. With the heating conditions and holding times, the coarse grains of specimen #2 were replaced by new and fine grains, with an increase in the number of crystal grains of 243. Compared with specimen #1, the number of crystal grains of specimen #2 increased by 29.9%. During cryogenic treatment, the grain size of specimens #3 to #5 was refined, with a change in cryogenic parameters. The number of specimen grains was 224, 235, and 304, which represented an increase of 19.8%, 62.6%, and 25.7%, respectively. 

**Figure 11:**
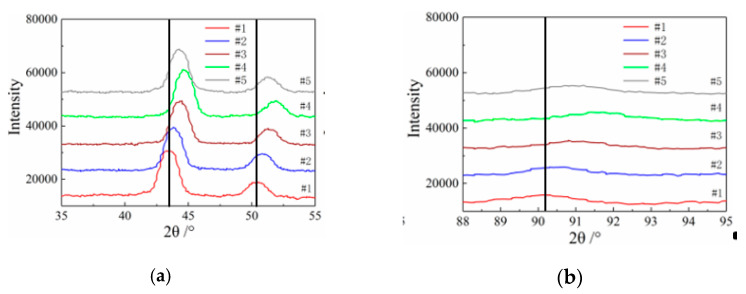
The diffraction peaks of the specimens for different cryogenic parameters. (**a**) 35°–55° diffraction peak distribution of specimens. (**b**) 88°–95°diffraction peak distribution of specimens.

For the cryogenic grain refinement, the diffraction peak patterns of the specimens in Figure 8 were amplified by selecting 2θ angles of 35°–55° and 88°–95° to observe the deviation in diffraction peaks for different specimens. As shown in Figure 11, after heat treatment and cryogenic treatment, there was a deviation in specimen diffraction peak patterns, which indicates that lattice distortion occurred inside the specimens, which led to a deviation in diffraction peak positions. The strain energy that was generated by the degree of subcooling in the cryogenic treatment caused lattice distortion, which sped up specimen nucleation [15,20]. In conclusion, the accelerated nucleation leads to more grains in the specimen, which results in a finer grain size.

The Hall–Petch formula:

(1) σs=σ0+kd−12

where $\sigma_{s}$ is the yield strength, $\sigma_{0}$ is the lattice friction, *d* is the grain diameter, and *k* is a material parameter.

According to Equation (1), $\sigma_{y}$ is influenced by the material grain size. A smaller grain size results in a larger material yield strength. Thus, the ability to resist specimen dislocation slip is enhanced, which results in a reduction in mismatch stress and affects the specimen macroscopic residual stress [21].

To investigate the variation in the dislocation density quantitatively, the density of geometrically necessary dislocation (*ρ*^GND^) was calculated according to the kernel average misorientation (KAM) in EBSD, and the calculated results were compared for each cryogenic parameter. KAM was the core of 24 nearest neighboring points, which was used to assign a scalar value to each point to indicate its local orientation difference.

The *ρ*^GND^ can be expressed by

(2) $$\rho^{\mathrm{GND}}=2KAM_{\mathrm{ave}}/\mu b$$

where μ is the step length that was selected for the EBSD test; b is the length of the Burgers vector; and $KAM_{\mathrm{ave}}$ represents the average KAM value of the selected region, which can be calculated by

(3) $$KAM_{\mathrm{ave}}=\exp\left[\frac{1}{N}\sum_{1}^{i}lnKAM_{L,i}\right]$$

where $KAM_{L,i}$ is the local KAM value at point *i*, and *N* represents the number of points in the test area.

$KAM_{\mathrm{ave}}$ is the average of the local orientation difference in the selected region θ, and thus Equation (3) can be simplified to

(4) $$\rho^{\mathrm{GND}}=2\theta /\mu b$$

**Figure 12:**
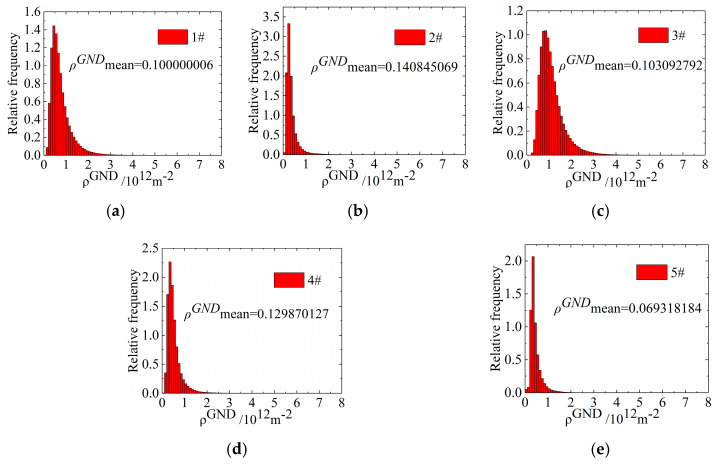
Specimen *ρ*^GND^ distribution for different cryogenic parameters. (**a**) Specimen #1. (**b**) Specimen #2. (**c**) Specimen #3. (**d**) Specimen #4. (**e**) Specimen #5.

The *ρ*^GND^ must be selected to characterize the variation in dislocations, and the *ρ*^GND^ distribution that was calculated for the specimens treated with different cryogenic parameters is shown in Figure 12. The calculated results are as follows: #1: *ρ*^GND^mean = 0.100000006 × 10^12^/m^2^, #2: *ρ*^GND^mean = 0.140845069 × 10^12^/m^2^, #3: *ρ*^GND^mean = 0.103092792 × 10^12^/m^2^, #4: *ρ*^GND^mean = 0.129870127 × 10^12^/m^2^, #5: *ρ*^GND^mean = 0.069318184 × 10^12^/m^2^. Compared with untreated specimen #1, the average *ρ*^GND^ was 0.100000006 × 10^12^/m^2^, and the average *ρ*^GND^ of the specimens increased to 0.140845069 × 10^12^/m^2^ after heat treatment. After cryogenic treatment, the average ρ^GND^ of specimens #3 to #5 decreased to 0.103092792 × 10^12^/m^2^, 0.129870127 × 10^12^/m^2^, and 0.069318184 × 10^12^/m^2^, respectively, which indicates that the dislocation density increased during heat treatment and decreased during specimen nucleation in cryogenic treatment.

In summary, the mechanism of cryogenic treatment to eliminate residual stress inside the specimen is summarized as two components. First, the strain energy that was generated by the subcooling degree in the cryogenic treatment led to lattice distortion and accelerated nucleation, which increased the number of nuclei and refined the grain size. Second, specimen grain refinement led to an increase in the grain boundary area, which led to an increase in the material yield strength, reduced dislocation slip at the grain interface, and reduced the residual stress inside the specimen by reducing the mismatch stress. Grain refinement caused by cryogenic treatment decreased the specimen dislocation density, and this process released part of the residual stress.

## 4. Conclusions

(1) After solid solution treatment, the internal residual stress of the hydrogen-resistant steel material showed a state of tensile stress in the core and a compressive stress on both sides. After cryogenic treatment, however, the spatial distribution characteristics of the residual stress remained stable, which is consistent with the initial state, but the stress amplitude decreased. Compared with the residual stress amplitude of untreated specimen #1, which was −350–270 MPa, after cryogenic treatment at 350 °C for 2 h, −130 °C for 10 h, and 350 °C for 2 h, the residual stress of the specimen was reduced by ~170–200 MPa, which is a reduction of ~57–63%.

(2) The physical phases of the hydrogen-resistant steel specimens were stable after different cryogenic treatments, and all were single-phase austenite without carbide precipitation. It was concluded that the reduction in the internal residual stress of the hydrogen-resistant steel specimens after cryogenic treatment was not caused by phase transformation or carbide precipitation.

(3) Cryogenic treatment resulted in obvious grain refinement and a reduction in the dislocation density, which was also the fundamental reason for the reduction in internal residual stresses in the specimens. Compared with untreated specimen #1’s grain number of 187, after cryogenic treatment at 350 °C for 2 h, −130 °C for 10 h, and 350 °C for 2 h, specimen #4’s grain number was 304. Compared with the single heat-treated specimens, the dislocation density of samples #3 to #5 after cryogenic treatment was reduced, which indicates that cryogenic treatment after heat treatment reduced the specimen dislocation density. After cryogenic treatment, specimen #2’s dislocation density was 0.140845069 × 10^12^/m^2^, and the dislocation density of specimen #5 was reduced to 0.069318184 × 10^12^/m^2^.

## Figures and Tables

**Table 1:** Chemical composition of hydrogen-resistant steel (mass fraction, %).

Alloy	C	Ni	Cr	Ti	Al	Mo	Nb	Mn	V	Si	P	S	Fe
HR-2	≤ 0.02	6	21	-	-	-	-	9	-	≤ 0.2	≤ 0.005	≤ 0.006	Bal.

**Table 2:** Aging treatment parameters.

Specimen Number	Test Parameters	Quantity
#1	untreated	3
#2	350 °C 4 h	3
#3	350 °C 2 h, −80 °C 10 h, 350 °C 2 h	3
#4	350 °C 2 h, −130 °C 10 h, 350 °C 2 h	3
#5	350 °C 2 h, −190 °C 10 h, 350 °C 2 h	3
